# Tribromphenol

**Published:** 1889-03

**Authors:** 


TRIBROMPHENOL.—Tribromphenol, prepared by shaking a solution of carbolic acid with bromine water, is one of the latest introductions for antiseptic purposes. We read in *Notes on New Remedies* that.

According to Dr. Grimm (*Apoth.-Zeit.*) a gauze containing two or three per cent. of tribromphenol, when saturated with blood serum or urine, remains perfectly odorless for fourteen days, while a one per cent. gauze only begins to smell after five days. One per cent. of an ammoniacal solution of tribromphenol added to the putrescent blood serum is said to destroy the bacteria in half an hour, while a half per cent. solution is effective in one hour. Its presence in a gelatin cultivation liquid in the proportion of 3 in 1,000 is said to prevent the development of putrefactive bacteria. This antiseptic is formed on shaking a solution of carbolic acid with bromine water, and occurs in white, soft, odorless needles, melting at 95° C. and subliming undecomposed at a higher temperature. It is soluble in alcohol, ether and chloroform, but is not readily soluble in glycerin, carbolic acid water or dilute alcohol It is readily soluble in caustic alkalies, from which it is separated unaltered by acids. When administered internally it is said to be taken up by the alkaline secretions of the intestinal canal and eliminated with the urine as tribromphenol-sulfuric acid.

